# Ultrastructural study of vitellogenesis of *Ligula intestinalis* (Diphyllobothriidea) reveals the presence of cytoplasmic-like cell death in cestodes

**DOI:** 10.1186/s12983-015-0128-7

**Published:** 2015-12-04

**Authors:** Aneta Yoneva, Tomáš Scholz, Daniel Młocicki, Roman Kuchta

**Affiliations:** Institute of Biodiversity and Ecosystem Research, Bulgarian Academy of Sciences, 2 Gagarin Street, 1113 Sofia, Bulgaria; Institute of Parasitology, Biology Centre of the Czech Academy of Sciences, Branišovská 31, 370 05 České Budějovice, Czech Republic; W. Stefański Institute of Parasitology, Polish Academy of Sciences, 51/55Twarda Street, 00-818 Warsaw, Poland; Department of General Biology and Parasitology, Medical University of Warsaw, 5 Chałubinskiego Street, 02-004 Warsaw, Poland

**Keywords:** Vitellogenesis, Ultrastructure, Paraptosis, Cestoda, Diphyllobothriidea, *Ligula intestinalis*

## Abstract

**Background:**

The tapeworm *Ligula intestinalis* (Diphyllobothriidea) is one of the most fascinating cestode parasites because it may cause parasitic castration of its second intermediate host, teleost freshwater fishes, due to inhibition of production of fish gonadotropic hormones. Large-sized (length up to 1 m) larvae called plerocercoids develop several months in the body cavity of freshwater fish and affect host behavior to facilitate transmission to the final host, a fish-eating bird. Vitellogenesis, i.e. formation of vitellocytes, is a key process in formation and nutrition of female gametes, oocytes in many flatworms, mainly parasitic Neodermata. The present study provides the first ultrastructural evidence in flatworms (Platyhelminthes) of the process that is interpreted as cytoplasmic-like cell death, i.e. a special case of programmed cell death (paraptosis) in vitellocytes of *L. intestinalis*.

**Results:**

As molecular markers for paraptosis are not yet available, its identification was based on morphological criteria. Electron microscopy analyses revealed evident structural changes in vitellocytes associated with progressive cytoplasmatic vacuolation, swelling of the granular endoplasmic reticulum and mitochondria. In addition, the present study has shown that vitellocytes of *L. intestinalis* share numerous features in common with the members of other earliest evolved eucestodes.

**Conclusions:**

The present study indicates that paraptotic-like cell death may occur in parasitic flatworms (Neodermata). The presence of GER-bodies in mature vitellocytes indicates close relationship between the Diphyllobothriidea, Caryophyllidea and Spathebothriidea, which are considered as the earliest evolved groups of the Eucestoda. Beyond the general similarities, however, a number of differences exist between the morphology, chemical composition and amount of these inclusions which could be due to the variations in their embryonic development, life cycle strategies and definitive host groups.

## Background

*Ligula intestinalis* (Linnaeus, 1758) (Cestoda: Diphyllobothriidea) is a worldwide distributed tapeworm of veterinary importance that affects cyprinid fishes impeding their reproduction by parasitic castration [1]. This species undergoes a complex three-host life-cycle including a freshwater planktonic copepod, a fish and a piscivorous bird that represents the definitive host [1]. The longest living stage in the life cycle represents the larval stage – plerocercoid, which grows several months or even years in the body cavity of the fish as second intermediate host and may reach up to 1 m and cause considerable host body deformation [1]. Reproductive organs of plerocercoids may reach almost full maturity except for production of eggs, which are formed and shed in the definitive hosts. Prepatent period, i.e. time from infection to production of eggs in the definitive host, is extremely short (around 2–3 days depending on temperature) as is longevity of adults in a bird host (just a couple of days).

Plerocercoids may influence behaviour of their fish hosts and significantly reduce their fecundity or even lead to castration [1–3]. This parasite-induced inhibition of production of gonadotropic hormon in infected fish is an interesting model for studies on host-parasite interface and parasitic castration. Even though the precise mechanism of host castration by plerocercoids of *L. intestinalis* has not yet been discovered, this tapeworm has also attracted attention because of detection of several genetic lineages that indicate the existence of cryptic species [4, 5]. Therefore, numerous studies focused mainly on parasite-host relationships have been carried out in the past decades (reviewed by [6]), but none of them has considered any aspect of the female reproductive system (unique for the euneoophoran Platyhelminthes in the presence of a vitellarium) participating in the egg formation [7, 8], which may represent a possible target for the development of novel therapeutics [9].

Recently, a series of ultrastructural studies on vitellogenesis in cestodes has been published, including two papers focused on species of the order Diphyllobothriidea [10, 11]. These studies provided some data on reproductive biology of these tapeworms, but their importance for assessment of the interrelations in diphyllobothriidean cestodes appeared to be limited by a low number of taxa studied. Therefore, vitellogenesis and vitellocyte ultrastructure of another species of this evolutionarily ancient cestode order (see [12]) were studied to supplement existing information.

## Methods

Adults of *L. intestinalis* (Linnaeus, 1758) were obtained from the intestine of the freshly killed great crested grebe *Podiceps cristatus* (Linnaeus) (Aves: Podicipediformes) collected in Záhlinice, North Moravia, Czech Republic on 20 April 2007 (vouchers deposited at the helminthological collection of the Institute of Parasitology, Biology Centre of the Czech Academy of Sciences in České Budějovice, Czech Republic; Coll. No. IPCAS C-150). Live worms were rinsed in 0.9 % NaCl solution and processed for transmission electron microscopy (TEM). Mature and gravid proglottids were separated and fixed with cold 2.5 % glutaraldehyde in cacodylate buffer. After 10 days in the fixative, samples were rinsed overnight in 0.1 M sodium cacodylate buffer at pH 7.4, postfixed in cold (4 C) 1 % OsO_4_ in the same buffer for 1 h, dehydrated in a graded series of ethanol and propylen oxid, embedded in Araldite and Epon and polymerized at 62 °C for 48 h. Ultrathin sections (60–90 nm in thickness) through selected regions were cut with diamond knife on a Leica Ultracut UCT ultramicrotome and placed on copper grids. Post-processing included staining with uranyl acetate and lead citrate according to Reynolds [13]. The observations were carried out using a JEOL 1010 transmission electron microscope at 80 kV (Laboratory of Electron Microscopy, Institute of Parasitology, Biology Centre of the Czech Academy of Sciences, České Budějovice, Czech Republic).

**Fig. 1:**
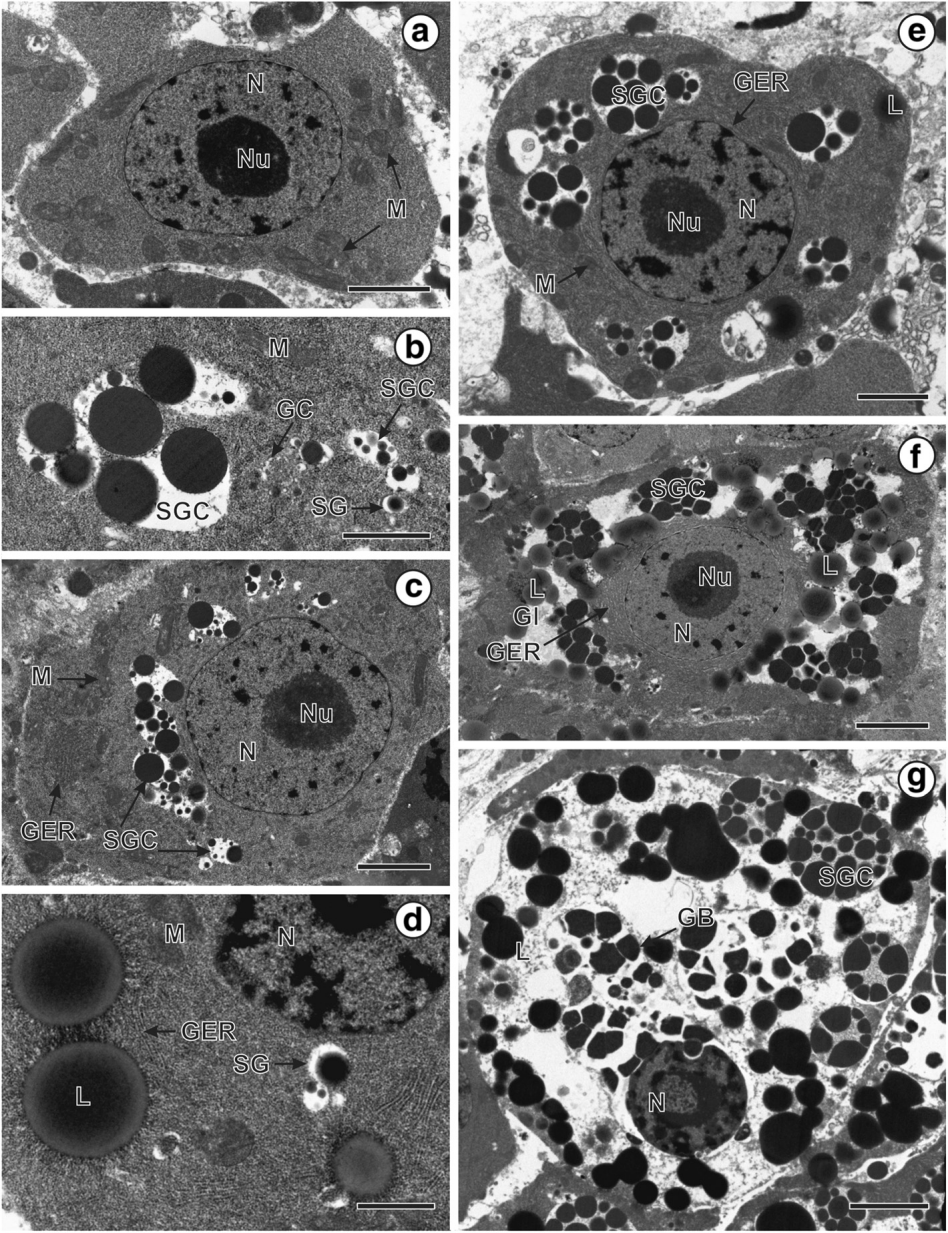
Consecutive stages of vitellogenesis in *Ligula intestinalis* and details of cytoplasmic inclusions. **a** An immature vitellocyte. **b** Formation of shell globule clusters. **c** Early stage of vitellocyte maturation. **d**, **e** Advanced stage of vitellocyte maturation. **f** Mature vitellocyte with numerous shell globule clusters and lipid droplets. **g** Mature vitellocyte with shell globule clusters, lipid droplets, GER-bodies and highly vacuolated cytoplasm. Note: progressive cytoplasmic vacuolation. Scale: **a**, **e**: 2 μm; **b**, **c**, **f**: 0.2 μm; **d**: 1 μm; **g**: 5 μm. *Abbreviations to all figures*: GB, GER-bodies; GC, Golgi complex; GER, granular endoplasmic reticulum; L, lipid droplet; M, mitochondria; N, nucleus; Nu, nucleolus; SG, shell globules; SGC, shell globule clusters; I, immature vitellocyte; II, early stage of vitellocyte development; III, advanced stage of vitellocyte maturation; IV, mature vitellocyte

## Results

### Immature vitellocytes (Figs. 1a, 3I, 4a)

Immature vitellocytes of *L. intestinalis* show high nucleus/cytoplasmic ratio (Figs. 1a, 4a). These are irregularly-shaped cells containing a large, spherical nucleus, a relatively small amount of moderately electron-dense granular, rich in free ribosomes cytoplasm and numerous scattered round to oblong mitochondria. Nucleoplasm contains electron-dense clumps of heterochromatin and a roundish electron-dense nucleolus (Figs. 1a, 3I).

### Early stage of differentiation (Figs. 1b, c, 3II, 4b)

The cytoplasmic content of vitellocytes at the early stage of differentiation is characterized by the presence of granular endoplasmic reticulum (GER), Golgi complex, spherical and oblong mitochondria. Ovoid nucleus contains a nucleolus and electron-dense patches of heterochromatin (Fig. 1c). Accumulation of mitochondria, GER and Golgi complexes were observed frequently nearby the newly formed shell globules (Fig. 1b). Preliminary aggregation of single globules into shell globule clusters takes place during this stage of vitellocyte differentiation (Figs. 1b, c, 4b). Early vitelline clusters are composed of 2–6 shell globules of different sizes (ca. 0.15–0.60 μm in diameter) (Fig. 1c). Shell globules are loosely packed and do not show arrangement into typical clusters.

### Advanced stage of maturation (Figs. 1d, e, 3III, 4c, d)

A few lipid droplets are formed in the cell cytoplasm, which is already filled with shell globule clusters during the advanced stage of vitellocyte maturation (Fig. 1e). They are first visible in the peripheral cytoplasm but later on appear in the electron-lucent cytoplasmic region filled with shell globule clusters (Figs. 1d, 4c, d). The clusters are composed of loosely packed electron-dense shell globules (ca. 2 μm in diameter). Individual shell globules within the clusters are relatively homogeneous in shape and electron-density. Only a few profiles of granular reticulum can be observed near the nuclear membrane of the spherical nucleus (Fig. 1d). Their nucleoli become more heterogeneous in nature following the maturation of vitellocytes (Figs. 1e, 4d ); zone of granular component and dense fibrillar component are clearly visible.

**Fig. 2:**
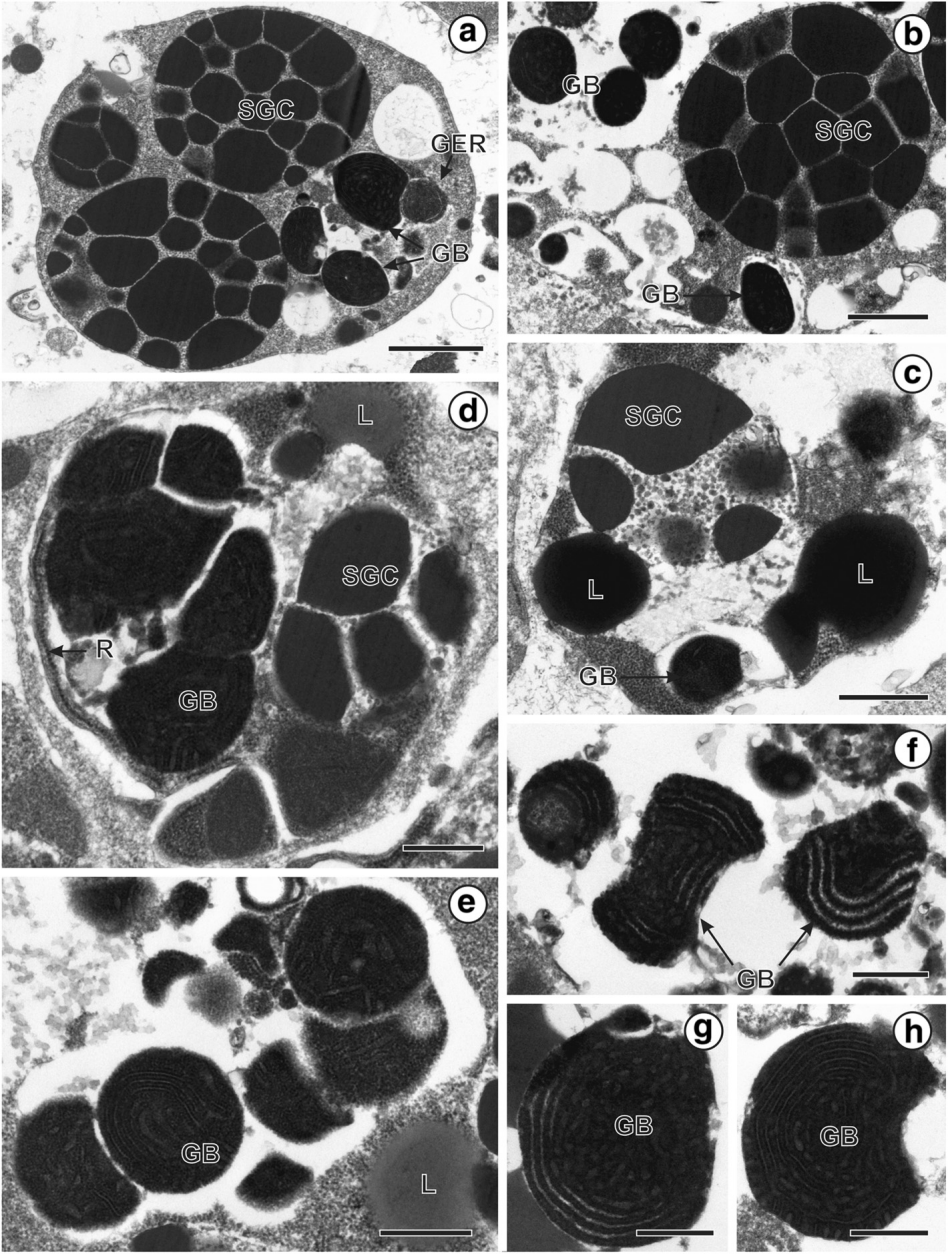
Ultrastructural details of shell globule clusters and GER-bodies in *Ligula intestinalis* vitellocytes. **a** Shell globule clusters and GER-bodies. **b**, **c**, **d**, **e** Large regions of focal cytoplasmic vacuolation containing GER-bodies and shell globule clusters. **f**, **g**, **h** High power magnification showing details of GER-bodies. Scale: **a**: 2 μm; **b**: 1 μm; **c**, **e**: 0.8 μm; **d**: 0.6 μm; **f**, **h**: 0.4 μm; **g**: 0.3 μm

**Fig. 3:**
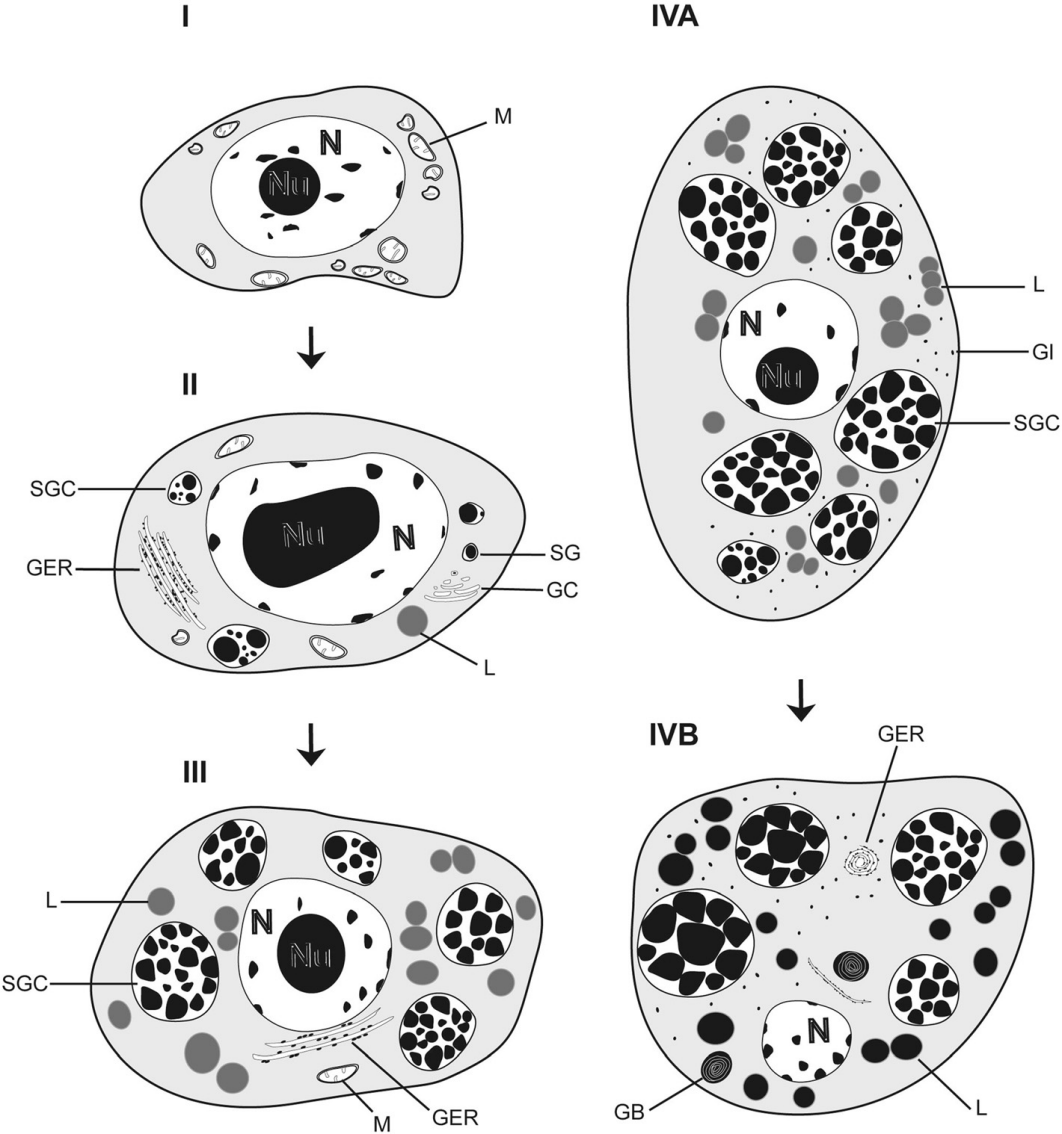
Schematic diagram of the cytodifferentiation of vitellocytes of *Ligula intestinalis* **. I**, immature vitellocyte; **II**, early stage of vitellocyte development; **III**, advanced stage of vitellocyte maturation; **IVA** and **IVB**, mature vitellocyte

**Fig. 4:**
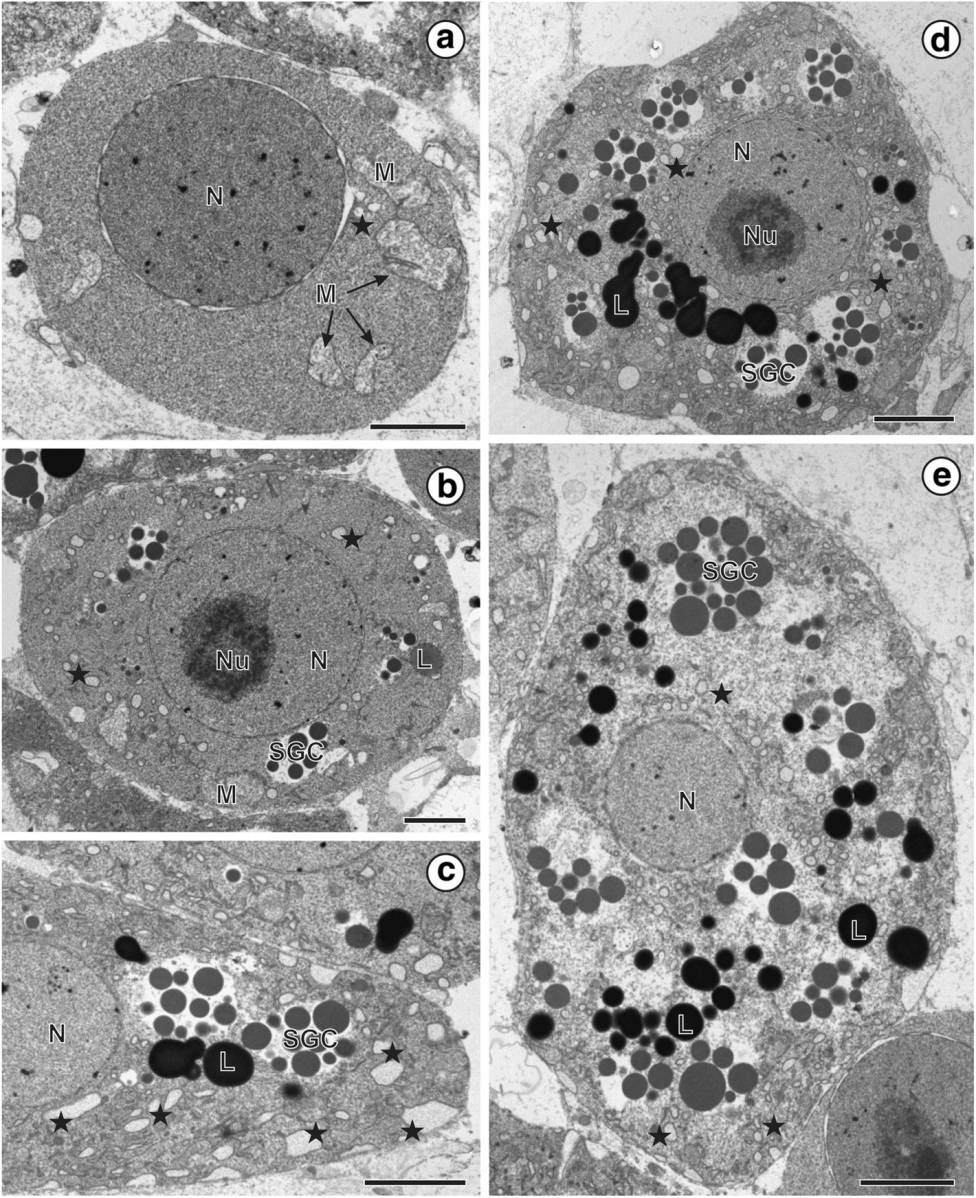
Paraptotic-like cell death during vitellogenesis of *Ligula intestinalis*. **a**–**e** Vitellocytes cytoplasm shows progressive cytoplasmic vacuolation, swollen mitochondria and granular endoplasmic reticulum (asterisks) – features characteristic for paraptosis. **b**, **d** The nucleus contains a large nucleolus and the chromatin is homogeneously dispersed. Note: the lack of apoptic characteristic of the nucleus in mature vitellocytes. Scale: **a**, **b**: 2 μm; **c**, **d**, **e**: 3 μm

### Mature vitellocytes (Figs. 1f, g, 2a–h, 3IVA, B, 4e)

The cytoplasm of mature vitellocytes contains shell globule clusters, lipid droplets, glycogen granules scattered between the lipid droplets and GER-bodies (Figs. 1f, g, 2a–h, 4e). Structure of mature vitellocyte cytoplasm indicates its gradual degradation. It becomes electron-lucent and all of the cytoplasmic organelles such as GER and mitochondia progressively disappear (Fig. 1g). A peripherally situated nucleus decreases in size in mature vitelline cells, but remains spherical. The most characteristic feature at this stage is the presence of GER-bodies (Fig. 2a–e) embedded in highly vacuolated cytoplasm. They are distributed mainly in the central cytoplasm, close to the nuclear membrane. GER-bodies are composed of spirally coiled granular endoplasmic reticulum (Fig. 2f, g, h) and are localized in close association to shell globule clusters, being frequently embedded in the same concentric regions of the focal cytoplasmic vacuolation (Fig. 2a–d). High power magnification (Fig. 2d) shows that they are composed of concentrically arranged membranes with membrane-bound rows of ribosomes on their cytosolic surface and less electron-dense GER cisternae (Fig. 2d, f, h).

Evident decrease in the number of lipid droplets and changes in their electron-density may be observed at this stage of vitellocyte differentiation (compare Fig. 1d, f and g). Simultaneously progressive cytoplasmic vacuolation, swelling of mitochondria and GER cisternae may be observed during the cytodifferentiaton of viltellocytes (Fig. 4a–e).

## Discussion

The process of vitellogenesis in *L. intestinalis* is in general similar to that of the other diphyllobothriidean species already studied, namely *Diphyllobothrium latum* (Linnaeus, 1758), *Cephalochlamys namaquensis* (Cohn, 1906), *Duthiersia expansa* Perrier, 1873 and *Schistocephalus solidus* (Müller, 1776) (see [10, 11]). This pattern includes the presence of four basic developmental stages of vitellocyte maturation and the presence of four types of vitelline inclusions, such as: shell globules/shell globule clusters, lipid droplets, glycogen granules and GER-bodies. Not surprisingly, most of these vitelline inclusions are very similar to those reported in other ‘lower’ (= early evolved) eucestodes with polylecithal embryonic development.

However, conspicuous differences exist in the form and number of vitelline inclusions, which may vary between species of the same dipyllobothriidean families [11]. This is likely due to diverse life cycle strategies of individual taxa, i.e. one or two intermediate hosts in the life cycle, aquatic vs. terrestrial habitat, different definitive host groups (amphibians, reptiles, birds and mammals), etc., with corresponding modifications of embryonic development and egg morphology [14].

In general, early stages of vitellocyte development in *L. intestinalis* showed no peculiarities, wherein immature and maturing vitellocytes have several features in common with that of other diphyllobothriidean cestodes, i.e. the cytoplasm matrix is abundant of cell organelles participating in synthesis and secretion of shell globules and the formation of shell globule clusters which take part in the egg-shell formation. However, there is high intraspecific variation in the size and number of shell globules and morphology of shell globule clusters.

In contrast, advanced and mature vitelline cells of *L. intestinalis* show differences when compared with those of other diphyllobothriidean species. The main differences lie in the type and amount of lipid droplets and the presence or absence of atypical vitelline inclusions.

A recent study on vitellogenesis in the Diphyllobothriidea by Yoneva et al. [11] has shown great variation in the chemical composition and amounts of lipid reserves accumulated in mature vitellocytes across the members of all three diphyllobothriidean families, which comprised species maturing in markedly different definitive hosts. All five diphyllobothriidean species studied contain different proportions of lipid droplets (saturated, unsaturated or both types) localized in the vitelline cytoplasm. The finding that amount, composition and distribution of lipids vary even between species of three diphyllobothriidean families raises an important question of their function.

Smyth and McManus [15] proposed two theories of the function of lipids in cestodes. They may play an important role as a source of energy or they may represent waste products of metabolism. According to these authors, the higher content of lipids in older proglottids supports the latter function. These authors observed such an increase also in *L. intestinalis* and *S. solidus* during their maturation. Młocicki et al. [16] and Bruňanská et al. [17] assumed that the increase of the lipid amounts in degenerating vitellocytes within intrauterine eggs of caryophyllidean cestodes *Wenyonia virilis* Woodland, 1923 and *Khawia sinensis* Hsü, 1935, parasites of freshwater teleosts, could represent the waste metabolic products of the early embryos. In contrast, reduced quantity of lipids in the cyclophyllidean cestode *Mosgovoyia ctenoides* (Railliet, 1890) (Anoplocephalidae) indicates that they may serve as energy reserves [18]. However, based on the results of the present study, we were unable to satisfactorily address the question whether lipid droplets in *L. intestinalis* represent essential nutritive reserve for the embryo or waste products of metabolism.

Glycogen is one of the major energy supplies in vitellocytes of cestodes. It occurs both as aggregates or rosettes (α-particles) for long-term storage and/or as discrete granules (β-particles) for rapid utilization. The type and amounts of glycogen reserves accumulated in the mature vitellocytes is highly variable in different group of cestodes, but β-particles apparently represent the most common form observed in cestode vitellocytes. The highest accumulation of glycogen was observed in the members of the order Caryophyllidea where high amount of glycogen granules was observed not only in their cytoplasm but also in the nucleus [19–21]. The accumulation of nuclear glycogen granules in vitelline cells of caryophyllideans is considered as a possible plesiomorphy of the Eucestoda [22].

Our observations also reveal that the amount of glycogen varies between diphyllobothriidean species ([10], present study). Vitellocytes of *D. latum* and *S. solidus*, which use homeotherm vertebrates as their definitive hosts, contain a significantly higher accumulation of glycogen granules compared to those of *D. expansa* and *C. namaquensis*, whose adults mature in reptiles and amphibians, respectively, i.e. poikilotherm definitive hosts [10, 11]. *Ligula intestinalis* matures in homeotherm definitive host (a fish-eating bird), but its vitellocytes contain only a small amount of glycogen granules randomly distributed throughout the vitelline cytoplasm. This indicates that the volume of glycogen in vitellocytes may not be always directly correlated with the type of definitive host.

Atypical subcellular structures (lamellar bodies/GER-bodies) seem to be present in most species of the earliest evolved eucestode orders, i.e. Caryophyllidea, Spathebothriidea and Diphyllobothriidea (see, e.g., [10, 23, 24]).

Our data on four species of diphyllobothriideans clearly show that the formation of atypical lamellar inclusions in the cytoplasm of mature vitellocytes coincides with the breakdown of the granular endoplasmic reticulum ([10, 11, 23], present study). They have been shown to consist of spherical areas of electron dense cytoplasm enclosed by concentrically rows of GER and thus they are not membrane-bounded structures. Based on these findings, a GER-dependent origin of lamellar structures in diphyllobothriidean cestodes can be assumed.

The same structures were also observed in the egg-enclosing vitellocytes of the caryophyllidean cestodes *W. virilis* and *K. sinensis* and those in the vitelloduct of another caryophyllidean from freshwater teleosts, *Caryophyllaeus laticeps* (Pallas, 1781) [17, 23, 25]. The role of GER-bodies in mature vitellocytes has not been fully investigated. It has been suggested that one of the most probable roles of GER-bodies is in the glycoprotein synthesis [23]. Furthermore, they may become remnants of GER and play a role in the formation of areas of focal cytoplasmic degradation. However, their function in vitellocytes is still far from being well known. Further investigation of the GER-bodies formation and structure should therefore provide useful information towards our understanding of their function and phylogenetic importance.

Various morphological changes were observed during differentiation of vitellocytes of *L. intestinalis* during the present study. Vitelline cells of *L. intestinalis* undergo extensive cytoplasmic vacuolation involving different structures and organelles. The appearance of vacuoles, GER and mitochondrial swelling in almost all cytoplasmic area resembles some of the characteristic morphological features of cytoplasmic cell death known as paraptosis, which is one of several types of programmed cell death (PCD) [26]. PCD is highly regulated process that occurs normally during development of multicellular organisms. Its function is to maintain tissue homeostasis by elimination of cells that are injured or no longer necessary. PCD includes different forms of active self-destruction, including an alternative non-apoptic form, named paraptosis. The term “paraptosis” was used for the first time by Sperandio et al. [27] to describe a morphologically distinct type of PCD that appears during development and neurodegeneration. In general, according to the original morphology-based definition, the most characteristic features of paraptosis are associated with cytoplasmic vacuolation as well as with the absence of nuclear fragmentation and chromatin condensation [27]. However, due to the lack of specific markers, paraptosis has not been carefully investigated and little is known about its mechanism. Therefore, its importance may have been underestimated and thus it is very important to improve our knowledge, especially that paraptosis can take place in certain pathological conditions, such as excitotoxicity, ischemia and neurodegeneration [28]. It is also believed that this type of PCD may be an ancestral form of programmed cell death, conserved across different forms of life [29]. Thus new data related to types of PCD other than apoptosis may be crucial for understanding evolution of PCD in general.

In the present study, based on morphological evidence, we hypothesize that vitellocytes of *L. intestinalis* during their development undergo changes, in order to achieve removal of non-functional cells, which might be considered as cytoplasmic-like cell death or paraptosis. This assumption is supported by the formation of characteristic paraptotic features, such as: cytoplasmic vacuoles (predominantly from the endoplasmic reticulum), GER and mitochondrial swelling, as well as the absence of characteristics typical for apoptosis, i.e., nuclear fragmentation, cellular blebbing and apoptic body formation. This is in agreement with observations made by Hoa et al. [30] who demonstrated that swelling of the GER and mitochondria is associated with a disruption of intracellular homoeostasis.

According to literature paraptosis is characterized by the presence of cytoplasmic vacuolation without nuclear fragmentation that begins with swelling of the endoplasmic reticulum and/or mitochondria [27, 28]. Apoptotic morphology and DNA fragmentation are absent. As mentioned above this characteristic was observed in our study during cytodifferentiation of *L. intestinalis* vitellocytes.

## Conclusions

Until now, there are no data that would indicate the existence of cytoplasmic cell-like death in flatworms (Platyhelminthes). Therefore, this is the first observation that provides ultrastructural evidence of cell changes that are interpreted as results of paraptotic-like cell death.

Our results may suggest that some of the cestode species (as *L. intestinalis*) may represent an interesting organism for studies focused on the understanding the process of cytoplasmic-like cell death. As this characteristic type of PCD may represent ancestral form of programmed cell death [29], its presence among such exceptional group as cestodes could help elucidate development and evolution of PCD in general.

Our results show that despite finding close morphological similarity among representative species of all diphyllobothriideans related with different host groups, no definite conclusion could be made regarding the possible phylogenetic implications due to the limited available data. However, the presence of atypical vitelline inclusions in mature vitelline cells, i.e. GER-bodies indicates close relationship between the Diphyllobothriidea, Caryophyllidea and Spathebothriidea, which are considered as the earliest evolved groups of the Eucestoda.
